# Editorial

**Published:** 1851-11

**Authors:** 


### THE MEDICAL EXAMINER.

PHILADELPHIA, NOVEMBER, 1851.

### EXPLANATION.

In the editorial comments which appeared in the September number of this Journal, in reference to the recent personal controversy between Drs. Robertson of South Carolina, and Ramsay of Georgia, we expressed the opinion "that there were grounds for attributing the attack upon our correspondent to personal pique, growing out of a family feud." This opinion was derived from statements made to us in letters received from Dr. Ramsay. Since the appearance of our remarks, Dr. Robertson has addressed us a communication in which he repels the "imputation" alluded to. We feel it our duty to record this denial, though our convictions remain unchanged as to the original character of the difficulty in question.

### NEW YORK MEDICAL TIMES.

Under the above title, the first number of a new Monthly Journal of Medicine was issued in New York, on the first of October last. J. G. Adams, M. D., is the Editor and Proprietor; and he announces that, in conducting it "while assistance from all quarters will be thankfully received, he designs to render it eminently a New York Journal, a reflection of what, in medical matters, New York does and thinks; a record of the proceedings of her Societies and Academy, of the cases of interest occurring in her Hospitals and Infirmaries."

The number before us is principally made up of valuable original matter, from Drs. Watson, Swett, Cocke, and other gentlemen connected with the New York Hospitals, and abundantly fulfils the editor's programme. He has our cordial wishes for the success of the '*Times*,' which certainly promises to be a most creditable addition to the periodical medical literature of the country.

### MEDICAL DEPARTMENT U. S. ARMY.

A board for the examination of Assistant Surgeons for promotion, and of candidates for admission, will meet in New York on the 15th instant.

The board will consist of Surgeon T. G. Mower, president; Surgeons Cuyler, Stirecke, and assistant Surgeon Simpson, members. They will remain in session for two or three weeks.

### MEDICAL SCHOOLS OF PHILADELPHIA.

The classes assembled at the various schools bid fair to equal, if not exceed, those of the last session, notwithstanding the many circumstances calculated to operate against them, in the shape of "hard times," short crops, &c.

### OBITUARY.

DIED, in Carroll County, Miss., on the 18th of June last, Dr. J. H. FITTS, aged twenty-five.

This is the second death we have had to record within the last few months from among our private pupils. Dr. Fitts, during a residence of eighteen months in this city, had gained many warm friends by his uniformly correct and manly deportment. He graduated with distinction at Jefferson Medical College, and received the certificate of merit from the Philadelphia Association for Medical Instruction. We desire to record his estimable qualities, because the example of those who die young is often lost to those who would most profit by it.

### LONDON MEDICAL EXAMINER.

We gratefully acknowledge the receipt of this excellent Journal, a notice of which had accidentally been omitted from our notices to Correspondents. It is among our most valued exchanges.

